# Hernia of the Ovary

**Published:** 1882-07

**Authors:** 


### HERNIA OF THE OVARY.

At a recent meeting of the Royal Medical and Chirurgical Society (*British Medical Journal*), Dr. Robert Barnes said that scanty advantage had been taken of the opportunity which the ovary, brought to the surface of the body, offers for physiological observation. He cited in abstract some of the most marked cases of hernia of the ovary which have been published, notably those of Goucy, Pott, Desault, Lallemand and Poquet, Deneux, Veboux, Cæsar Hawkins, Oldham, Holmes Coote, Œttingen, Meadows and Lawson, Courty, Leopold, Beigel, Boinet, Rheinstädter and Raffo, and related two cases observed by himself. 1. The patient, a single woman, aged 41, had always enjoyed good health. At 24 she sustained a rupture in the left groin and wore a truss; at 38 she observed a second swelling behind the first. The swelling and tenderness of the ovary were observed before and during the menstrual periods. Corresponding sphygmographic observations showed distinct rise of tension preceding the flow, and subsiding when the flow set in. The ovary was removed. A description and illustration of it were submitted by Dr. Goodhart. Dr. Barnes referred to Dr. Chambers' case in the *Obstetrical Transactions*, in which bodies simulating ovaries turned out to be testicles. He discussed the etiology of hernia of the ovary and uterus, citing Cruveilhier's views. He referred to the frequent complication of anomalies of development of the genital organs in association with hernia of the ovary; also with extra-uterine gestation. He enumerated the varieties of hernia of the ovary, and referred to the supposed greater frequency of inguinal hernia when the ovary was concerned; to the greater frequency of congenital hernia; the complications with intestine and epiploon; the dependence of hernia of the uterus upon pre-existing hernia of the ovary, citing Cruveilhier's theory and the confirmatory conclusions of Puech, Deneux, and Cæsar Hawkins. He then discussed physiological points, illustrated by the observations of the herniated ovary; how the ovary swelled concurrently with increased tension of the vascular system before menstruation; how the round ligaments swelled. He discussed the order in which the phenomena of menstruation occurred, arguing that the ovarian nisus was the *primum mobile*, that nervous and vascular tension followed, and lastly, the menstrual flow; resting greatly upon sphygmographic observations. He suggested that the recent practice of oöphorectomy on Battey's principle would supply opportunities for deciding this and other questions; and proposed that sphygmographic observations should be made upon the subjects of this operation. He then discussed the diagnosis and treatment of hernia of the ovary, contending that it furnished a legitimate motive for Battey's operation *quoad* this affection at least. The paper was illustrated by sphygmographic tracings, by drawings of the amputated ovary, and by a cast of the parts.

Dr. Routh said that such cases as those described by Dr. Barnes were important, not only as indicating the symptoms of ovarian hernia, but as contributions to the history of menstruation, which no doubt began in the ovary. He had, however, observed some symptoms to which Dr. Barnes had not referred, in cases in which the ovary had fallen into Douglas' pouch; especially on the left side. In one case, that of a young lady, who had a displacement of the ovary into the left side of Douglas' pouch, where it had become adherent, pressure on the ovary caused sexual excitement. This, if observed in hernia of the organ, would be an additional means of diagnosis. Another symptom observed in these displacements was, that pressure on the ovary produced a feeling of sickness like that arising from pressure on the testicle.

Mr. Hulke said that cases of hernia of the ovary were rather frequently met with by surgeons. Of course, care was necessary in making a diagnosis. In 1871, 38 cases of hernia of the ovary were recorded and classified by Englisch of Vienna (see *Biennial Retrospect* of New Sydenham Society, 1871-72). Of these, in 27 the hernia was inguinal; and in 9 of these the displacement was on both sides. In most of the inguinal cases the hernia was congenital. As regarded the absence of any process of peritoneum in one of Dr. Barnes' cases, this might have existed and have become atrophied. In congenital cases, it appeared that the ovary was almost always accompanied by the Fallopian tubes; while, when the hernia was acquired later in life, the ovary generally came down alone. In many of the recorded cases, a distinct tubular process of peritoneum was described. As regarded operation, each case must be judged on its own merits. He had seen cases where a lightly fitting truss produced no discomfort to the patient, while in others it could not be borne.

Mr. Langton referred to the difficulty of diagnosis. At the Truss Society, among more than 4,000 cases of inguinal hernia in women, there were 67 cases of hernia of the ovary. Of these, 42 were congenital, and in 25 the displacement occurred at various ages. The ovary was reducible in 29 of the congenital cases, and in 27 of these the patients were quite relieved by the use of a truss; the other two did not report themselves, and were probably cured. Of the cases in which the hernia occurred later, 8 were reducible and 17 irreducible; the proportion being the reverse of that in congenital hernia. The conditions of the ovary during the menstrual periods varied. In some, there was swelling of the ovary with effusion of fluid, which became absorbed; but when the hernia had been first observed between the ages of one and twelve, no such excitement of the ovary was developed. The application of a truss gave relief in most cases; in none were there any indications for operation.

Dr. Heywood Smith said that it was very rare to find sexual excitecitement produced by pressure on the ovary; pressure generally caused pain of a sickening character. Might not the sexual excitement in Dr. Routh's case have been caused by irritation of the pudic nerve, in consequence of the adhesions to the pelvis?

Dr. Barnes thought that the remarks of Mr. Hulke and Mr. Langton tended to confirm the opinion of Cruveilhier, that most cases of hernia of the ovary were congenital, and that the displacement was most frequent on the left side. He had not met with any cases in which distinct sexual excitement was produced by pressure on the ovary. He thought that the greater frequency of displacement in Douglas' pouch on the left side was caused by the round ligament and the ovary being more lax there than on the right.—*Med. and Surg. Reporter.*

